# Adversarial Resolution Enhancement for Electrical Capacitance Tomography Image Reconstruction

**DOI:** 10.3390/s22093142

**Published:** 2022-04-20

**Authors:** Wael Deabes, Alaa E. Abdel-Hakim, Kheir Eddine Bouazza, Hassan Althobaiti

**Affiliations:** 1 Department of Computer Science in Jamoum, Umm Al-Qura University, Makkah 25371, Saudi Arabia; adali@uqu.edu.sa (A.E.A.-H.); khbouazza@uqu.edu.sa (K.E.B.); hmthobaiti@uqu.edu.sa (H.A.); 2 Computers and Systems Engineering Department, Mansoura University, Mansoura 35516, Egypt; 3 Electrical Engineering Department, Assiut University, Assiut 71516, Egypt; 4 Laboratoire d’Informatique et des Technologies de l’Information d’Oran (LITIO), University of Oran, Oran 31000, Algeria

**Keywords:** ECT, image reconstruction, deep learning, CGAN, ARE-ECT

## Abstract

High-quality image reconstruction is essential for many electrical capacitance tomography (CT) applications. Raw capacitance measurements are used in the literature to generate low-resolution images. However, such low-resolution images are not sufficient for proper functionality of most systems. In this paper, we propose a novel adversarial resolution enhancement (ARE-ECT) model to reconstruct high-resolution images of inner distributions based on low-quality initial images, which are generated from the capacitance measurements. The proposed model uses a UNet as the generator of a conditional generative adversarial network (CGAN). The generator’s input is set to the low-resolution image rather than the typical random input signal. Additionally, the CGAN is conditioned by the input low-resolution image itself. For evaluation purposes, a massive ECT dataset of 320 K synthetic image–measurement pairs was created. This dataset is used for training, validating, and testing the proposed model. New flow patterns, which are not exposed to the model during the training phase, are used to evaluate the feasibility and generalization ability of the ARE-ECT model. The superiority of ARE-ECT, in the efficient generation of more accurate ECT images than traditional and other deep learning-based image reconstruction algorithms, is proved by the evaluation results. The ARE-ECT model achieved an average image correlation coefficient of more than 98.8% and an average relative image error about 0.1%.

## 1. Introduction

During the 1980s, based on the computed tomography (CT) technique of medical images, researchers proposed electrical capacitance tomography (ECT) [1]. Because of its low cost and accuracy, ECT has been widely used in industrial process monitoring in reactors, pipelines, and containers, and wherever non-conductive components of a dielectric nature can be used. Knowing the internal distribution of materials inside an industrial process container or pipe is essential in many applications. Tomography plays a very important role in several industrial fields. Typical examples of the use of this technology include the food industry, industrial tomography, biomedical processes [2], gas–fluid flow [3], chemical and pharmaceutical processes [4,5], and non-destructive evaluations of invisible objects in dams and flood embankments [6].

The electrical capacitance tomography (ECT) can be defined as the use of electrodes to measure capacitance changes that are transformed into two-dimensional images as visual outputs using image reconstruction algorithms [7]. Typically, the electrode numbers in the ECT sensor controls the number of independent capacitance measurements (usually 28 to 496) and the acquisition rate varies from a few up to several thousand images per second [8]. Then, one or more high-performance PCs collaborating together, using mathematical models, can process the collected data and implement dedicated image reconstruction algorithms to make the appropriate diagnostic decision to effectively process control and automation [7].

The ECT can be implemented both in real-time [9] and offline mode [10]. The choice of the image reconstruction algorithm has a crucial role in the ECT process since it has a direct impact on the image quality [11]. The ECT image reconstruction process can be implemented through iterative algorithms, e.g., iterative Landweber method (ILM) [12], Newton Raphson [13], and Tikhonov regularization [14], as it can also be implemented through non-iterative methods, such as linear back projection (LBP) [15]. The speed and the simplicity of the non-iterative methods was not an argument for wide use because, in the same time, they suffer from deformations in the reconstructed images [16]. In comparison, iterative methods can generate higher quality images. They are, however, computationally very expensive, thus, more useful for offline processing. The need for tools that can compromise the trade-off between high quality reconstructed images and computational efficiency, is currently the main interest of machine learning (ML) [17,18], more specifically, deep neural network (DNN) methods [19]. DNN methods have been utilized in many fields due to their ability to map complex nonlinear functions [20,21]. DNN algorithms have been transferred and adapted such as in image reconstruction methods based on the convolutional neural network (CNN) [22], multi-scale CNNs [23], long short-term memory (LSTM) [24], and autoencoder [25]. To solve the forward problem and to estimate the capacity measures, Deabes et al. used a capacitance artificial neural network (CANN) system [26,27]. Thanks to its ability to effectively use specific geometric relationships hidden in commonly used unstructured grid models, the authors in [28] proposed to use the graph convolutional network(s) (GCN), to increase the quality of the ECT image. Moreover, a long short-term memory image reconstruction (LSTM-IR) algorithm was implemented to map the capacitance measurements to accurate material distribution images [24].

Generative adversarial networks (GANs) are very interesting techniques that have been recently developed in ML [29,30]. These networks allowed obtaining new results that were previously thought to be difficult to achieve: text to image generation [31], text generation in different styles [32], generation and defense against fake news [33], conversion of sketches to images [34], generation of photo-realistic images [35], and even game designs learned by watching videos [36]. The conditional generative adversarial network (CGAN) [37], which is a particular version of the standard GAN, allowed better control over the output of generative adversarial models. Subsequently, this kind of GAN was applied in medicine to the CT of soft tissues [38] as well as to tomography of the structure of materials with synchrotron radiation [39,40].

A novel post-processing adversarial resolution enhancement (ARE-ECT) model for ECT reconstructed image quality improvement is proposed in this paper. The proposed model is inspired by the deep learning networks for image super-resolution [41,42]. Principally, we assumed that a CGAN can be trained to enhance the reconstructed low-resolution ECT images from few capacitance measurements. Particularly, a CGAN is trained in generator and discriminator networks to produce high-resolution images from lower resolution reconstructions. As a result, when trained with pairs of ECT image reconstructions of a simulated phantom and a phantom itself, the CGAN model learns how to enhance the resolution of the inputs. Accordingly, the proposed adversarial model achieves better results than the recent complex, time-consuming non-linear ECT image reconstruction methods, and brings the reconstructed images closer to the phantom reference quality.

The contributions of this paper can thus be summarized as follows:

- The adversarial resolution enhancement (ARE-ECT) model was developed in the problem of the ECT image reconstruction quality improvement.
- The proposed model aimed to predict enhanced ECT image reconstructions from the lower quality ones.
- Our CGAN-based approach produces qualitative and quantitative improved results in ECT image resolution better than current complex and time-consuming non-linear reconstruction algorithms.

The remainder of this paper is organized as follows: Section 2 covers the ECT image construction problems. Section 3 describes the DNN models, including GAN and CGAN. Section 4 introduces a new ARE-ECT model to enhance the ECT image construction. Section 5 describes the dataset used to train, test, and evaluate the proposed model. Section 6 discusses the experimental results and the validity of the proposed model. Finally, Section 7 presents our conclusions.

## 2. Problem Statement

**Figure 1:**
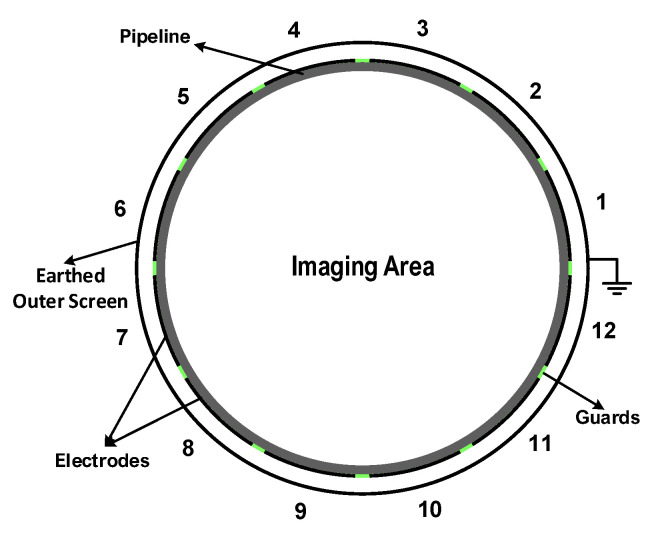
ECT system with 12 electrodes.

The ECT problem is a typical image reconstruction problem. Particularly, given input data measurements, a higher resolution image is to be reconstructed. The input measurements could be any input data that are correlated to the reconstructed image. The modalities of the input data do not necessarily have to be the same of the output data. In the ECT problem, the input data are a few sensor reading numbers that are fed into the reconstruction algorithm as the input signal. The ECT sensor generates readings via a number of electrodes (n=12), which are evenly mounted around the imaging area. Figure 1 illustrates the sensor setup. To capture the variations in the permittivity of the inner distribution, the mutual capacitance of each pair of these electrodes are measured independently [43]. This pairwise measurement process results in a total number of capacitance measurements of M=n(n−1)/2. To keep the uniformity in the electric field, decrease the external coupling, and eliminate any interference, the electrodes are separated by insulating guards [44].

The distribution of the permittivity of the inner material within the area of interest affects the distribution of the electric field, which is defined according to the Poisson linear partial differential equation, as shown in Equation (1).

(1) ▽·(ε(x,y)▽ϕ(x,y))=−ρ(x,y),

where ε(x,y) is the distribution of permittivity, ϕ(x,y) is the potential distribution, and ρ(x,y) denotes the charge distribution.

The mutual capacitance between electrode pairs is given by Equation (2).

(2) $$C_{uv}=\frac{Q_{v}}{▽V_{uv}}=-\frac{1}{V_{uv}}\oint_{\Gamma_{v}}\varepsilon \left(x,y\right)▽\phi \left(x,y\right)\cdot \hat{k}\;dl$$

where $C_{uv}$ identifies the mutual capacitance between two electrodes *u* and *v*, $Q_{v}$ denotes the charge on the sensing electrode, which is defined according to the Gaussian law, ▽Vuv denotes the potential difference, $\Gamma_{v}$ represents a closed path embracing a detection electrode, and $\hat{k}$ stands for a unit vector normal to $\Gamma_{v}$.

The ECT image reconstruction involves solving two types of problems: the forward and inverse. The forward problem refers to the numerical computation of the capacitance measurements from the sensor reading, according to Equation (3):

(3) $$C_{M\times 1}=S_{M\times N}\left(\varepsilon_{0}\right)\cdot G_{N\times 1}$$

where *C* is the calculated capacitance, *S* is the sensitivity matrix, *N* = 16,384 is the number of image pixels, and *G* is the permittivity distribution. The sensitivity matrix is the Jacobian of the capacitance with respect to pixels evaluated at $\varepsilon_{0}$.

The ECT inverse problem refers to estimating the permittivity distribution, *G*, given the capacitance measurement, *C*, and the sensitivity matrix, *S*. A non-iterative solution can be obtained directly from Equation (3) using non-iterative algorithms, e.g., LBP, as shown in Equation (4).

(4) $$G=S^{T}C$$

However, the obtained images using such a paradigm suffer from poor quality. This shortcoming could be dealt with using iterative algorithms, e.g., the Landweber algorithm (LW), as shown in Equation (5).

(5) $$G_{k+1}=G_{k}-\lambda S^{T}\left(SG_{k}-C\right)$$

where λ is the relaxation parameter, $SG^{k}$ is the forward problem solution, and *k* is the iteration number. However, despite the significant improvement achieved in the reconstructed images quality, it comes with high computational costs.

## 3. Deep Neural Network Models

The ECT reverse problem can be looked at as a data generation problem, which is controlled by certain constraints. Specifically, a low-resolution input image is the control input that governs the creation of the higher resolution permittivity solution. Over the years, many models have been developed based on DNNs. One of the most popular models that is extensively researched and applied in image processing and computer vision is the generative adversarial network (GAN). In addition, a conditioned version called CGAN was developed to control the reconstructed image and guarantee high quality outputs [45]. Therefore, we propose using a CGAN model for this purpose. In the following subsections, we provide a brief overview of GANs and CGANs. Then, we describe the proposed ECT image reconstruction model using CGAN.

### 3.1. GAN

GAN [29] was introduced to force two competing learning agents to enter a performance race during data generation. The first agent, which is the generative model *G*, is responsible for capturing the data distribution. It learns how to generate from scratch data patterns that follow the same distributions of input data. The second agent, which is the discriminative model *D*, learns how to discriminate between real data samples drawn from the input data and the fake data samples that are generated by *G*. During the training process, each agent optimizes for its own objective function simultaneously in a competitive manner. This leads to a state, in which the generated data by the generator is hardly identified as fake.

In the training process, *G* learns a distribution $p_{g}$ over the input data. This is accomplished by building a mapping function from a noise distribution to a generative data space $G(z,\theta_{g})$. The discriminator *D* learns how to generate a Boolean decision indicating whether its input data come from training data or generated by *G*. The purpose of the training process is to adjust parameters for the generator to deceive the discriminator by minimizing log(1−D(G(z))). At the same time, the parameters of the discriminator are adjusted to optimally detect the real data by maximizing log(D(x)). These two competing objectives are aggregated in a combined objective value function V(G,D), as show in Equation (6).

(6) $$\begin{array}{c} V\left(G,D\right)=\arg\min_{G}\max_{D}\left(\underset{x∼p_{data}\left(x\right)}{E}\left[\log\left(D\left(x\right)\right)\right]+\underset{z∼p_{data}\left(z\right)}{E}\left[\log\left(1-D\left(G\left(z\right)\right)\right)\right]\right) \end{array}$$

### 3.2. CGAN

GAN has been modified and developed into many variants over the last few years. CGAN is one of these models [37]. The new thing about this model is labeling the data during the training process. Table 1 shows the differences between these two models. It may look similar, yet the major difference between them involves adding additional information to control the output [46,47]. So, the CGAN is an extension of the generative adversarial networks, which include a condition to both the generator (*G*) and discriminator (*D*) by feeding some extra information, *y*, into the input layer as an additional constraint. This extra information helps guide both *G* and *D* by incorporating auxiliary data from the same or other modalities. For the objective function of Equation (6), this turns out to condition *G* and *D*, as shown in Equation (7).

(7) $$\begin{array}{c} V\left(G,D\right)=\arg\min_{G}\max_{D}\left(\underset{x∼p_{data}\left(x\right)}{E}\left[\log\left(D\left(x|y\right)\right)\right]+\underset{z∼p_{data}\left(z|y\right)}{E}\left[\log\left(1-D\left(G\left(z\right)\right)\right)\right]\right) \end{array}$$

## 4. ARE-ECT Model

**Figure 2:**
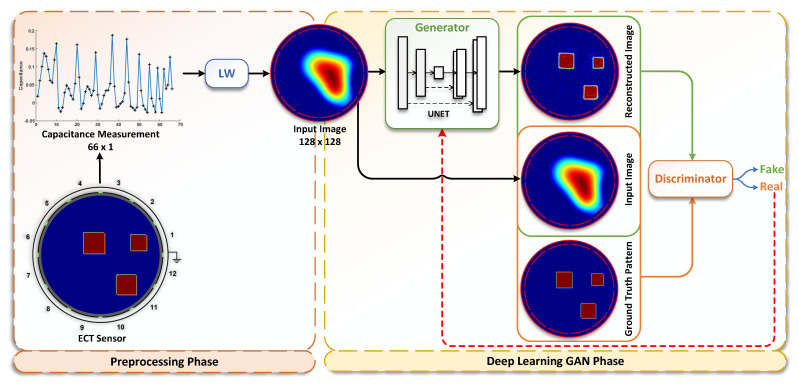
Architecture of ARE-ECT model.

**Figure 3:**
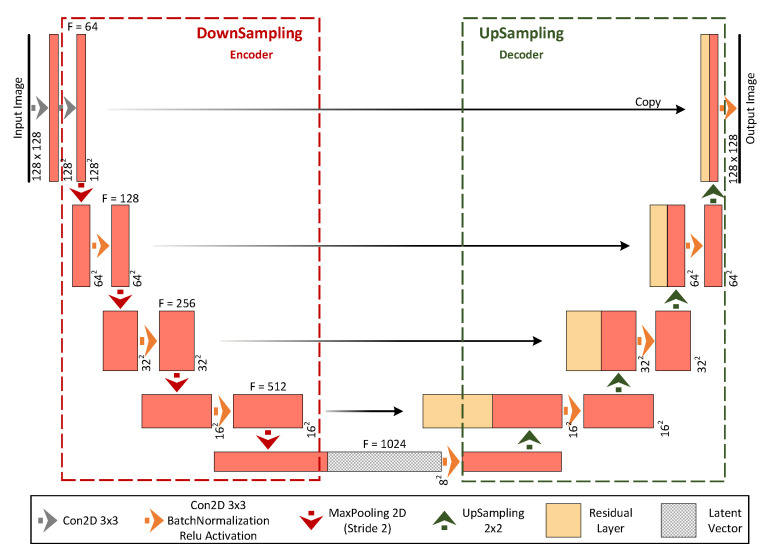
The architecture of the used UNET network in the generator.

As explained in Section 2, the main objective of the ECT image reconstruction problem is to generate a high quality permittivity distribution image, given a lower resolution distribution input image. Therefore, the first step of the proposed ARE-ECT model is to prepare the input image for the generator operation. This preparation is performed in a preprocessing phase, as shown in Figure 2. The input to this preprocessing phase is the capacitance reading set. The ECT capacitance sensor produces a 1×66 raw vector data, i.e., M=66. Afterwords, the input image is generated using traditional LW of Equation (5) with k=0. The initial image of the permittivity distribution is provided by some fast matrix multiplication. The input image resulted from the preprocessing phase is fed to a generator. This generator could be a traditional autoencoder. However, although autoencoders are capable of reconstructing such patterns, the spatial information of the input signals are not modeled with sufficient accuracy. Given that the spatial information of the inner distributions is essential for the reconstruction of the flow pattern image, another generator that can preserve such spatial representation is mandatory. UNet is a good candidate to satisfy this requirement [48]. Therefore, we adopted UNet to construct the flow pattern in the generator module. Figure 3 illustrates the details of the used UNet in ARE-ECT. Four blocks were used on the encoder side, and similarly, four blocks were placed on the decoder side. The latent vector size was eight. The input layer’s low resolution image, generated by the preprocessing phase, was concatenated with the generated image by the final layer. Similarly, each input of the hidden layers on the decoder side was concatenated with the output of the corresponding layer from the encoder side.

The UNet generator module produces a flow pattern, which is considered a fake sample for the discriminator training. A synthetic data generator was developed to generate real samples, $FP_{r}$, for the purposes of discriminator training. As shown in Figure 2, the architecture of our UNet generator was designed with two sections: down- and upsampling. The main idea of UNet is to map a low resolution input image at a size of 128×128 to a 1-D vector and then reconstruct it back to a high quality image. The contraction of the downsampling (encoder) applies a 3×3 convolutional layer, batch normalization, and Relu activation followed by a 2×2 max pooling in each step. This stage generates a downsized image of a size equal to 64×64 with 128 features, and it continues to the latent vector size of 8×8 with 1024 features. The layers at the decoder (upsampling) section employ a 2×2 upsampling layer after convolution. During the upsampling process, the corresponding feature maps from the downsampling part are reused to reduce the distortion of images. They are appended directly after the upsample layer. The proposed model is designed for a 12-electrode ECT sensor setup. If any change in this setup, in terms of the number of sensors occurs, a new dataset must be generated. Therefore, every generated dataset is valid only for its underlying hardware configuration. This is because the resolution of the initially generated low-resolution images varies with the number of installed sensors.

## 5. ECT Dataset

**Figure 4:**
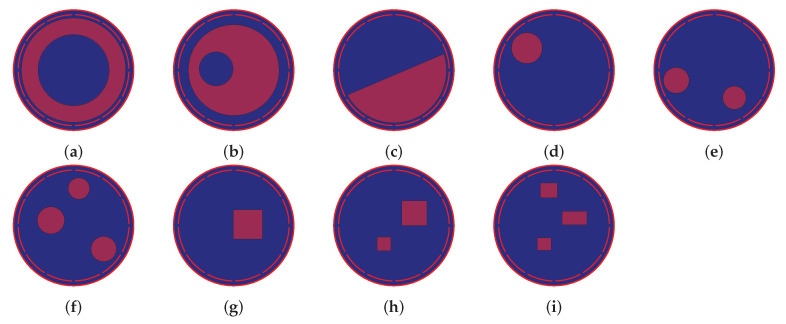
Samples of different flow patterns. (**a**) Ring, (**b**) annular, (**c**) stratified, (**d**) 1 cir. bar, (**e**) 2 cir. bars, (**f**) 3 cir. bars, (**g**) sq. bar, (**h**) 2 sq. bars, (**i**) 3 sq. bars.

We implemented a MATLAB GUI software package to build different configurations of ECT sensors. Various flow patterns can be simulated and their forward problems can be solved to generate the corresponding capacitance measurements. An extensive ECT benchmark dataset was developed for training and testing of the proposed ARE-ECT. A traditional image reconstruction algorithm was used to reconstruct the permittivity distributions, which used the initial image *x* for the deep learning ARE-ECT model. In this paper, we used the LW algorithm as the inversion algorithm to generate the initial input image. The dataset consisted of 320 k samples, each one was a pair of an actual permittivity distribution vector as a ground truth, and the reconstructed image of the LW algorithm corresponding to each capacitance measurement vector. The sizes of the actual distribution, and the LW reconstructed image were 128×128 = 16,384. The ECT sensor was composed of 12 electrodes as shown in Figure 1. The sensor pipe was made from PVC material with a relative permittivity of 2. The diameter and the thickness of the pipe was 100 and 2 mm, respectively. The electrodes were separated by gaps of 4 degrees, and the span angle of each electrode was 26 degrees. The dataset contained five different flow patterns, 10 k ring patterns, annular with 20 k patterns, 10 k stratified patterns, 1–3 circular bars with 140 k patterns, and 140 k patterns of 1–3 square bars. Figure 4 shows some samples of various flow patterns from the generated ECT dataset. The low phase was air with a relative permittivity value equal to 1, and the relative permittivity of the high phase glass was (4). Random variables were used in building the dataset. For instance, a uniform random variable with a range of 10% to 95% of the imaging area’s radius was applied to the ring’s width of the annular flow. The stratified flow height was assigned to a uniform random variable in a range of 5–95% of the diameter of the sensing field. The number of circular and square bars varied from 1 to 3. The generated data have some discrepancies in the number of instances within each type to reflect varying degrees of randomness. Additionally, every flow pattern had a different number of attributes that determined its geometric specifications. For instance, the attributes that characterized a ring flow pattern were just two—the inner and outer radii, while those of the square bar patterns were the number of bars, their lengths, widths, and planner locations. This large attribute dimensionality variation implies consequent large variations in the number of generated instances that represented the input data space.

## 6. Experimental Results and Analysis

The ARE-ECT model was trained and tested by using the developed ECT datasets. The overall network’s performance of the proposed algorithm was verified based on the reconstruction results of the testing dataset. Typically, the ARE-ECT model was validated during the training phase to avoid overfitting; 10% of the training samples were randomly chosen as a validation set. The more comprehensive the data simulation, the stronger the generalization performance of the model after training. Therefore, the generalization ability of the proposed model was tested using a testing dataset, generated phantoms that were not included in the training dataset, and practical experimental data.

### 6.1. Validation Metrics

Typically, the relative image error (IE) and correlation coefficient (CC) between ground truths and reconstructed permittivity distributions were applied to evaluate the image quality and the reconstruction algorithm’s performance [7]. The relative IE is defined as Equation (8).

(8) $$IE=\frac{||G-G^{*}{\left|\right|}_{2}}{{\left||G|\right|}_{2}}$$

where $G^{*}$ represents the reconstructed image from the ARE-ECT model, and *G* represents the original distribution.

The similarity between the reconstructed image and the ground truth image was measured by CC, which is defined in Equation (9)

(9) $$CC=\frac{\sum_{i=1}^{N}\left(G_{i}-\bar{G}\right)\left(G_{i}^{*}-{\bar{G}}^{*}\right)}{\sqrt{\sum_{i=1}^{N}{\left(G_{i}-\bar{G}\right)}^{2}\sum_{i=1}^{N}{\left(G_{i}^{*}-{\bar{G}}^{*}\right)}^{2}}}$$

where $\bar{G}$ and ${\bar{G}}^{*}$ are the mean values of *G* and $G^{*}$, respectively. *N* = 12,932 is the number of pixels in the imaging area.

The ARE-ECT model was designed and trained using the Python TensorFlow machine learning platform [49], and Keras deep learning API [50]. The testing process was carried out using the reconstructed image from LW as input to the ARE-ECT model, while the output was the reconstructed permittivity distribution. The testing set contained 96 k samples; hence, the ARE-ECT performance was evaluated by the mean values of the IE and CC. The smaller the relative IE and the bigger the CC, the better the performance.

### 6.2. Qualitative Results on Simulation Test Dataset

A simulation testing dataset that had been unseen by the network during the training process was used to validate the reconstruction ability of the proposed ARE-ECT model. Typically, the developed ECT dataset containing 320 k pairs was divided into a 70% (224 k pairs) training dataset and a 30% (96 k pairs) testing dataset. The training and testing datasets are quite different since the dataset for each flow pattern was randomly generated.

**Figure 5:**
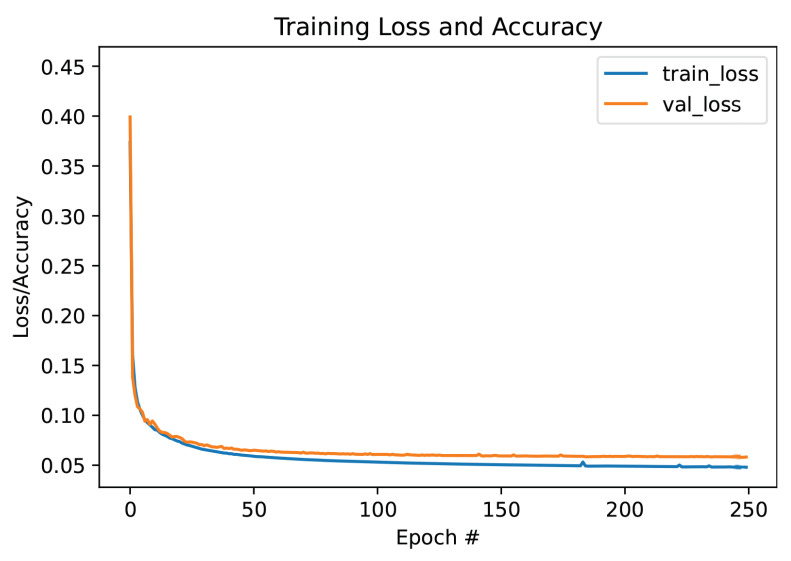
Training and validation loss curves.

The loss curve, shown Figure 5, declines over 250 epochs on the training and validation sets. The minimum, maximum, and average values of relative IE and CC of the testing dataset are stated for each flow type in Table 2. The results prove that the ARE-ECT model can reconstruct images that are very close to the ground truth distributions. The average values of the relative IE = 0.1019 and CC = 0.9884 show a significant overall performance of the ARE-ECT model when applying the LW input images.

**Figure 6:**
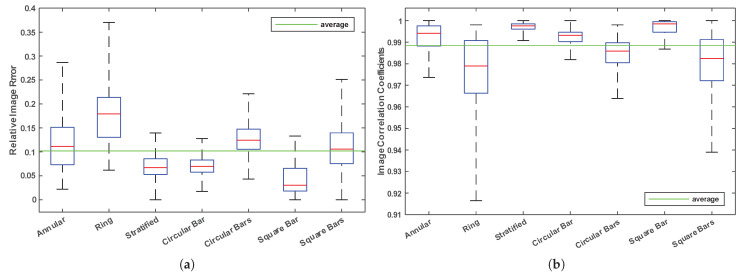
Box plots of testing criterion. (**a**) Relative image errors (ie), (**b**) correlation coefficients (CC).

The IE and CC for all flow types are drawn as box plots, Figure 6a,b, respectively.

Figure 6a,b show the substantial performance of the ARE-ECT model since 95% of the IE and CC are in reasonable intervals. From Table 2, the performance of the ARE-ECT model on the ring flow type is the lowest compared with other flow types. A single square bar flow type has the best results of relative IE, while for CC, annular, stratified, single circular, and square bar are more than 99%.

**Figure 7:**
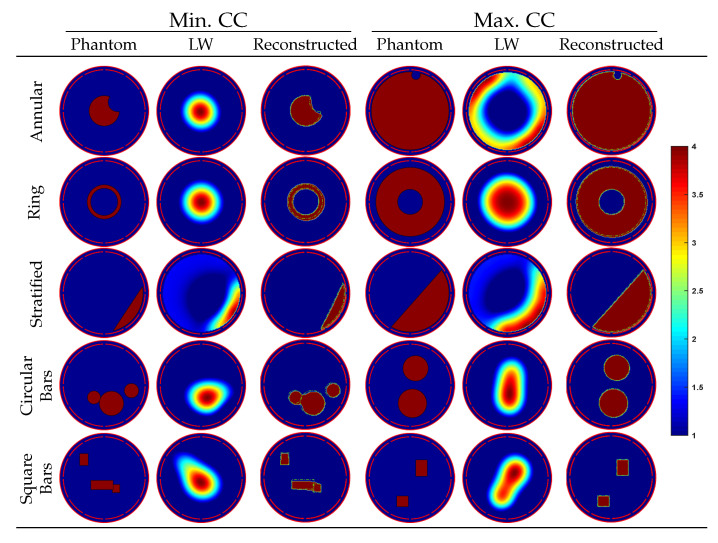
Examples of maximum and minimum CC image reconstruction results.

Reconstructed image instants equivalent to the minimum and maximum CC of each flow group in Table 2 are given in Figure 7. Visually, the reconstructed images with minimum CC, still very close to the ground truth permittivity distributions, and the reconstructed images with the maximum CC, obviously have better visual effects. The reconstructed images, shown in Figure 7 are almost the same as their ground truth distributions. For multiple circular and square bars, the reconstructed positions of objects are consistent with the true distributions. In general, our model performs well on the test dataset and has a strong ability to reconstruct images of all typical flow types with permittivity values of objects predicted correctly.

**Figure 8:**
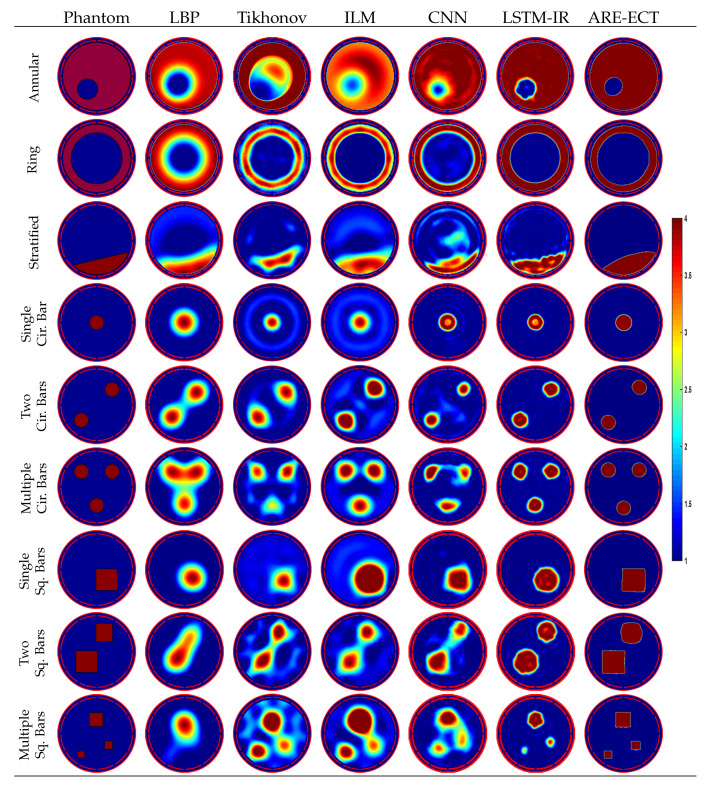
Reconstructed images of well known image reconstruction algorithms.

The performance and the reconstructed image qualities of the proposed ARE-ECT algorithm and other state-of-the-arts ECT image reconstruction algorithms are compared. An assortment of flow patterns have been set up to test the generalization ability of the proposed model. Figure 8 shows the compassion results, where the real phantoms are shown in the first column, and the reconstructed images from the LBP, iterative Tikhonov, ILM, CNN [22,23], LSTM-IR [24], and ARE-ECT algorithms are contained in the other columns, respectively. The hyperparameters of the Tikhonov and ILM algorithms were selected empirically. The optimal regularization parameter was selected, 0.01, while the iteration numbers of the Tikhonov and the ILM were 200 and 1000 iterations, respectively. The CNN algorithm is based on a multi-scale dual-channel convolution kernel composed of a dual-channel frequency division model [23], where each channel has five convolution layers. The CNN model is trained using the results of the LBP as inputs. The results of the ARE-ECT model have high image quality and accuracy with sharp object boundaries when compared to the reconstructed images from the LBP, iterative Tikhonov, ILM, and CNN algorithms. Visually, in Figure 8, the ARE-ECT model can reconstruct objects in the imaging area with sharp edges since there is no transition region between the reconstructed objects compared with the other algorithms. The generated objects have blurred zones around it, which increases the relative IE. Moreover, the results stated in Table 3, which are the IE and CC of the reconstructed images from the ARE-ECT model compared with the other algorithms, prove that the performance of the ARE-ECT model is better than other reconstruction algorithms.

### 6.3. Testing Results of Non-Existing Phantoms in Training Dataset

**Figure 9:**
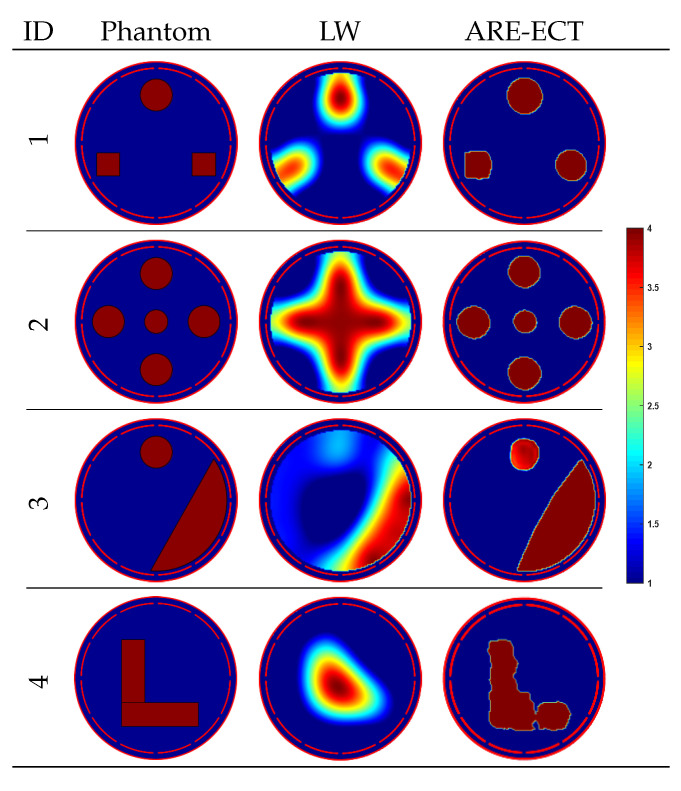
Image reconstruction results of phantoms not in training dataset.

New two-phase flow patterns, which are not included in the training dataset, were created to measure the generalization ability of the proposed ARE-ECT model. Four different flow distributions, from 1 to 4, shown in first column of Figure 9, were inputted to the trained ARE-ECT model. Relative IE and CC are listed in Table 4. Although none of these patterns exist in the training set, the ARE-ECT still can reconstruct them with high quality results. Although the ECT suffers from the inhomogeneous sensitivity map problem across its cross-sectional sensing domain, the reconstructed image of the five-bars phantom proves the ability of the ARE-ECT model to reconstruct phantoms located in the low and high sensitivity areas of the ECT sensor. The results are acceptable, although the reconstructed result is not quite sharp. The angles of the square object in the first sample and the L_Shape of the fourth sample are more rounded.

### 6.4. Evaluation Using Experimental Data

**Figure 10:**
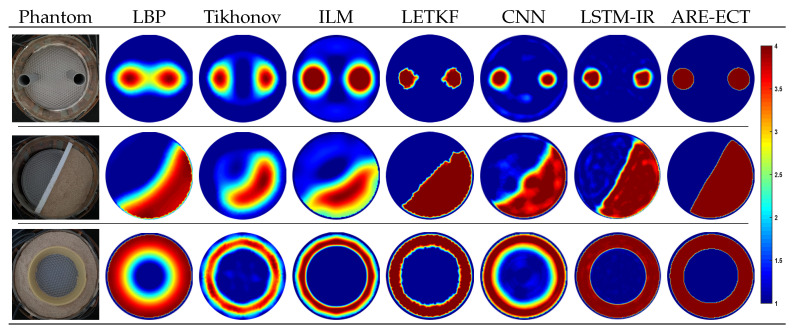
Experimental Setup and Reconstructed Frames.

The generalization ability of the ARE-ECT model was also measured by applying experimental data. Capacitance measurements from three two-phase flow types as the training set were generated as real testing inputs. The experiments were carried out using electrical capacitance volume tomography (ECVT) hardware system [51]. There were 36 channels in the ECVT to measure the capacitance among 12 electrodes ECT sensor with an imaging rate of 120 images/s. Static phantoms were placed in an imaging area with a radius of 140 mm surrounded by 12 electrodes. As shown in the first column of Figure 10, the bubble flow type was experimented by placing two plastic rods of radius r=20 mm inside the imaging area, while one-half of the imaging area filled with plastic particles (ϵ=4) simulated the stratified flow type. Filling a ring shape around the center of the ECT sensor with the plastic particles represented the annular flow type.

Figure 10 demonstrates the real distributions and the generated images from LBP, iterative Tikhonov, ILM, local ensemble transform Kalman filter (LETKF) [18], CNN, LSTM-IR, and ARE-ECT algorithms. The reconstructed images by the ARE-ECT model have high accuracy and sharp edges separate the two phases compared with the other reconstruction algorithms. Moreover, the ARE-ECT reconstructed images have fewer artifacts, much better visual quality, and are faster than that of the LBP. ARE-ECT is more efficient than traditional iteration algorithms, such as the iterative Tikhonov, ILM, and the LETKF, which can obtain good imaging quality but are still slow. Comparing the reconstructed images from the proposed ARE-ECT model with the other deep learning (DL) models, such as CNN and LSTM-IR, proves the potential of the proposed method in generating significant high quality images with accurate permittivity values and sharp boundaries. The core component of our method is CGAN, which exhibits stronger enhancement and resolution, increasing capabilities, compared to conventional DL methodologies. As the target problem model in this work is image enhancement, it is natural for our method to obtain benefits of the inherited capabilities of CGAN in this aspect. Moreover, since the UNet conditions the output side by input data, this further strengthens the enhancement capabilities of the proposed method.

### 6.5. Computational Time Measure

Typically, the performances of image reconstruction algorithms are evaluated by the imaging speed. For the experimental ECT data, Table 5 contains the imaging costs of different reconstruction algorithms. The algorithms were run on a PC with an i9 CPU (3.6 GHz) and 32 GB memory. The reconstruction time of the proposed model was 0.046 s, which was >135x, >115x, and >28x faster than ILM, iterative Tikhonov method, and LETKF, respectively. The ARE-ECT model was also faster than other DL models, and it constructed more accurate images compared to all other methods. The LBP was faster than our proposed method, but the image qualities were worse than our model. The imaging speed of the ARE-ECT model can also satisfy online application, as the LBP algorithm.

## 7. Conclusions

In this paper, a new ARE-ECT model based on the CGAN deep neural network was proposed to enhance the resolution of the ECT reconstructed images. The generator was built using UNet. For evaluation purposes, a big dataset was developed. It contained simulation data of 320 k capacitance measurements–flow image pairs for training, validating, and testing. For generalization and feasibility of ARE-ECT, data instances, to which the model was not exposed during the training phase, were included in the evaluation dataset. The experimental results proved the superiority of the proposed ARE-ECT over the state-of-the-art, both quantitatively and qualitatively. Efficiency evaluation results showed that ARE-ECT succeeded in beating existing high-quality methods in terms of execution speed by ’several tens of times’, particularly from 28× to 135×. Briefly, ARE-ECT achieved better performance than the computationally-expensive methods, yet with the same execution time order of the low-resolution reconstruction method, e.g., the well-known LBP. In terms of the overall generalization, the ARE-ECT exhibited good capabilities. Hopefully, the work presented herein will inspire researchers in the ECT field to further investigate other deep learning-based approaches to reconstruct the flow patterns in the sensing field of the multi-phase flow.

## Figures and Tables

**Table 1:** GAN vs. CGAN.

	GAN	CGAN
Input	Latent vector	Random and auxiliary data
Output	Classify as real or generated	Classify labeled data as real or generated
Type	Unsupervised	Supervised
Data	No control over data	Conditional data

**Table 2:** Minimum and maximum of relative IE and CC of testing results.

Flow Patterns	Min. IE	Max. IE	Average IE	Min. CC	Max. CC	Average CC
Annular	0.0219	0.2864	0.1160	0.9736	1.0000	0.9921
Ring	0.0562	0.3704	0.1781	0.9165	0.9980	0.9770
Stratified	0.0000	0.1395	0.0694	0.9907	1.0000	0.9970
Single Cir. Bar	0.0173	0.1276	0.0712	0.9819	1.0000	0.9923
Multiple Cir. Bars	0.0308	0.2178	0.1288	0.9639	0.9985	0.9845
Single Sq. Bar	0.0000	0.1329	0.0415	0.9868	1.0000	0.9965
Multiple Sq. Bars	0.0000	0.2512	0.1086	0.9390	1.0000	0.9803
Total Average	IE		0.1019	CC		0.9884

**Table 3:** IE and CC values of different ECT image reconstruction algorithms.

	Flow	LBP	Tikhonov	ILM	CNN	LSTM-IR	ARE-ECT
Relative Image Error (IE)	Annular	0.2412	0.1950	0.3351	0.1222	0.0561	0.0687
	Ring	0.3776	0.1216	0.2984	0.2107	0.0989	0.0941
	Stratified	0.2590	0.6953	0.3203	0.3365	0.2032	0.1994
	Cir. Bar	0.3923	0.6562	0.6575	0.2224	0.1420	0.0821
	2 Cir. Bars	0.4568	0.6638	0.4038	0.3274	0.1445	0.0990
	3 Cir. Bars	0.6083	0.7492	0.4275	0.4765	0.2043	0.0940
	Sq. Bar	0.3677	0.5841	0.6575	0.2490	0.2122	0.0991
	2 Sq. Bars	0.4988	0.3449	0.3294	0.3176	0.2415	0.1653
	3 Sq. Bars	0.5112	0.6070	0.6909	0.4999	0.2558	0.0528
Correlation Coefficient (CC)	Annular	0.8701	0.8885	0.9084	0.9590	0.9913	0.9864
	Ring	0.8110	0.9792	0.9576	0.9396	0.9857	0.9870
	Stratified	0.9126	0.4232	0.9100	0.8200	0.9401	0.9587
	Cir. Bar	0.6964	0.7754	0.7974	0.8860	0.9541	0.9850
	2 Cir. Bars	0.6681	0.8565	0.7963	0.8060	0.9640	0.9823
	3 Cir. Bars	0.5498	0.5652	0.7625	0.7325	0.9363	0.9862
	Sq. Bar	0.8442	0.8264	0.6575	0.8997	0.9277	0.9850
	2 Sq. Bars	0.7041	0.8326	0.8663	0.8527	0.9161	0.9617
	3 Sq. Bars	0.5099	0.6361	0.5688	0.6668	0.8707	0.9951

**Table 4:** Results of phantoms not in training dataset.

Phantom	IE	CC
1	0.2601	0.9049
2	0.1847	0.9427
3	0.2761	0.8909
4	0.2816	0.8852

**Table 5:** Reconstruction time in sec.

LBP	Tikhonov	ILM	LETKF	CNN	LSTM-IR	ARE-ECT
0.026	5.326	6.245	1.310	0.085	0.052	0.046

## Abbreviations

List of nomenclature and abbreviations

CT	Computed Tomography
ECT	Electrical Capacitance Tomography
ARE-ECT	Adversarial Resolution Enhancement
ILM	Iterative Landweber Method
LBP	Linear Back Projection
ML	Machine Learning
DNN	Deep Neural Networks
DL	Deep Learning
CNN	Convolutional Neural Network
LSTM	Long Short-Term Memory
CANN	Capacitance Artificial Neural Network
GCN	Graph Convolutional Networks
GAN	Generative Adversarial Network
CGAN	Conditional Generative Adversarial Network
LW	Landweber Algorithm
IE	Image Error
CC	Correlation Coefficient
LSTM-IR	Long Short-Term Memory Image Reconstruction
LETKF	Local Ensemble Transform Kalman Filter
ECVT	Electrical Capacitance Volume Tomography
